# Examining EFL teachers’ changing conceptions of research: A case study of a continuing professional development program in mainland China

**DOI:** 10.3389/fpsyg.2022.933061

**Published:** 2022-09-22

**Authors:** Yan Kang, Luxin Yang

**Affiliations:** ^1^English Education Department, College of Foreign Languages, Capital Normal University, Beijing, China; ^2^School of English and International Studies, The National Research Centre for Foreign Language Teaching Materials, Beijing Foreign Studies University, Beijing, China

**Keywords:** continuing professional development, conceptions of research, teacher change, EFL teachers, teaching and research

## Abstract

Drawing on teacher interviews, written teacher reflections, teacher research proposals, and research papers, this study explored the outcome and process of teacher learning during their participation in a continuing professional development program. It has been found that the teachers changed their conceptions of research over the course of learning concerning the nature, purpose, and process of research and the relationship between teaching and research. Dialogic exchanges and reading research papers, along with the scaffolding of the teacher educator, enabled the teachers to validate their practices, link others’ perspectives up to their own, and re-situate research in light of their current practices. The findings provide insights into the nature of teachers’ conceptual change and how learning opportunities can be better built into continuing professional development programs.

## Introduction

Continuing professional development has been a focus for curriculum development and teacher education research since the 1970s. There has been an extensive body of literature that explores the issue in terms of both theory and policy. In recent years, an increasing number of studies have sought to examine empirically teachers’ experiences of learning in such activities. However, few studies evaluate actual programs in specific contexts (Kiely and Davis, 2010; Borg, 2018). Researchers argued that high-quality continuing professional development programs should be designed based on a thorough understanding of what enables teachers to change and how teachers change as a result of learning in such programs (Borko et al., 2010; Sahin and Yildirim, 2015; Labone and Long, 2016; Tabatabaee-Yazdi et al., 2018; Hayes, 2019). Therefore, more studies need to probe into the program procedures that support teacher learning.

To address this need, the present study followed up on a group of Chinese school EFL teachers in a continuing professional development (CPD) program organized by the local EFL Teaching and Research Office (TRO) in a district of Beijing, China, with the aim of introducing reading–writing-integrated instructional principles (Hirvela, 2016) and improving the participating teachers’ teaching effectiveness through classroom research. Designed and implemented by the second author, an experienced teacher educator who specializes in EFL teaching and teacher education, the CPD program consisted of 4 monthly workshops, totaling 24 hours. The design of the program was based on two central assumptions about teacher learning. First, learning is effective when teachers share experiences and jointly explore their practices in collaborative processes. The collaborative dialog constitutes the social context that supports the construction of professional knowledge. Second, learning is furthered by reading research articles relevant to teachers’ teaching and research contexts. Therefore, this study in particular examined whether changes took place in teachers’ conceptions of research as a result of their participation in the CPD program and how the teachers utilized the learning opportunities afforded by the program to (re)construct their conceptions.

## Literature review

### Teachers’ conceptions of research

Since the 1960s, there has been an emergent drive to engage teachers more fully in educational research to improve classroom teaching and learning and, to a broader extent, promote their autonomous professional development. The underlying rationale is that when teachers engage with (through reading) and in (by doing) research, they will be able to not only make pedagogical decisions informed by sound research evidence but also play an ever more active role in curriculum development (Stenhouse, 1975; Hargreaves, 2001; Borg, 2010). Borg (2010) defined teacher research as “systematic inquiry” conducted by teachers in their professional contexts and pointed out that engagement in research can enhance teachers’ understanding of their work and, consequently, has the potential to promote quality teaching and learning in individual classrooms and inform institutional improvement and educational policy (p.395).

Stimulated by this interest in encouraging teachers to become research-engaged, researchers in the field of general education began to examine teachers’ conceptions of research such as what they think of research, the specific meanings they attach to research, and how such understanding influences their professional life (Shkedi, 1998; Everton et al., 2000, 2002). In the past decade, the same strand of inquiry began to emerge in the literature on English language teaching. One underpinning argument has been that efforts to promote teacher research engagement are more likely to be rewarded if they are based on an understanding of teachers’ conceptions of research and the role played by research in their work (Borg, 2009).

These studies, echoing those conducted outside ELT, revealed that English teachers worldwide held different conceptions of research, with some adhering to conventional positivist notions of scientific inquiry and others equating research with a teaching-oriented process; they often read or wrote less formal research articles than teaching-oriented articles that share innovative classroom activities and teaching approaches; their research engagement was largely driven by institutional requirements or considerations of promotion, rather than their own need for professional development (e.g., Allison and Carey, 2007; Borg, 2009; Gao et al., 2011; Gao and Chow, 2012; Vu, 2021; Gironzetti and Muñoz-Basols, 2022). Current research also revealed some barriers to teacher research, including lack of time, lack of awareness about the importance of research, lack of research skills, lack of mentorship, limited funding and resources, non-collaborative school culture, leadership attributes, and political issues (Barkuizen, 2009; Gao and Chow, 2012; Borg, 2013, 2010; Borg and Liu, 2013; Rahimi and Weisi, 2018; Farsani and Babii, 2019; Sato and Loewen, 2019; Alhassan and Ali, 2020; Li and Zhang, 2022). It has been suggested that teachers would be more inclined to change their conceptions about research, be better able to cope with the different constraints in their contexts, and consequently become more autonomous in their professional development when they were supported in their research engagement either by their colleagues or university teacher educators (Banegas et al., 2013; Wang and Zhang, 2014; Yuan and Lee, 2015; Dimmock, 2016; Cornelissen et al., 2017).

### Continuing professional development

It has been widely acknowledged that continuing professional development (CPD) plays an important role in improving or changing teachers’ cognition and practices. Kelchtermans (2004) defined CPD as a learning process that leads to “changes in teachers’ professional practice (actions) and in their thinking about that practice” (p.220). The term is adopted in the present study as it distinguishes the development of teachers throughout their career from their professional development during the teacher education and induction phases and connotes “a broader range of developmental possibilities” than one-shot in-service training that targets the transfer of certain content to teachers (Wermke, 2011, p. 666). Current research on continuing professional development tends to follow two thematic lines: one that focuses on the process of learning and the other that focuses on the product. The former targets the dynamics of knowledge construction, whereas the latter examines the implications of CPD activities for knowledge building, for example, the conditions that afford changes.

Teacher change in CPD programs is widely reported in general education research (Park and So, 2014; Ottley et al., 2015; Turner et al., 2017; Zimmerman et al., 2017). However, the volume of such research is limited in the ELT context (Borg, 2011). Within that small number of studies, the results however were quite mixed, with some reporting stability (Lamb, 1995; Kubanyiova, 2006; Sim, 2011; Ming, 2019), some providing evidence of significant change (Lamie, 2004; Kiely and Davis, 2010; Teng, 2016; Karimi and Zade, 2018; Gorter and Arocena, 2020; He and Bagwell, 2022), and others identifying different levels of change across individuals (Tillema and van der Westhuizen, 2006; Borg, 2011; So, 2013; Chaaban, 2016; Coburn and Borg, 2022). Borg (2011) pointed out that the impact of language teacher education on teachers deserves much more empirical attention as our understanding of the issue remains incipient. Therefore, additional research is needed to identify the conditions under which professional development opportunities are connected to teacher change.

Differences exist in these studies regarding program characteristics, research methodology, and how change is operationalized. Nevertheless, existing studies do reveal some conditions that lead to effective professional development. In a review of 38 professional development studies, Kalinowski et al. (2019) highlighted three broad areas of effective program characteristics: (1) structural features such as multiple delivery formats, expert involvement, and consideration of teachers’ needs, interests, and existing knowledge; (2) content-related features such as application-oriented knowledge; and (3) didactic features such as cooperation and collaboration, input, application and reflection, active learning, and supplementary materials. These features were consistent with previous research findings that argue for practice- and inquiry-based collaborative programs that characterize such components as peer observations, experience sharing, and meaningful discussions (e.g., Garet et al., 2001; van Veen et al., 2012; Park and So, 2014).

Drawing on data from interviews and written reflections, Kiely and Davis (2010) found that the collaborative component of the CPD program worked well with the teachers as they understood the context of collaboration as supportive and reassuring and based on shared values. However, the researchers found that the use of reading was not as successful as it had been expected, which they attributed to a lack of space for reading in teachers’ working lives and limited relevance of the reading to teachers’ daily practices. Likewise, Park and So (2014) proved the effectiveness of such activities as peer observations, individual reflections, and peer interactions in a school-initiated collaborative professional development program. In a study with Irian EFL teachers, Tabatabaee-Yazdi et al. (2018) proved that CPD components that lead to teachers’ success include the opportunities to update their knowledge, collaborate with others, reflect on teaching, and make important decisions in their classrooms.

Regarding the research on the change process, there are a relatively small number of studies that capture the dynamics of knowledge construction during teacher learning (e.g., So, 2013; Selvi and Martin-Beltrán, 2016; Choi, 2015, 2017; Ming, 2019). Adopting a sociocultural perspective, the researchers postulated that teacher change is a process of development or learning achieved by teachers as learners working in a learning community, and an outcome of complex interactions between the individuals and the various agents in that community, including supervisors, colleagues, teacher educators, and students (Clarke and Hollingsworth, 2002). Therefore, rather than equating “change” with a radical shift, the current study acknowledges the cumulative nature of teacher change and defines the teacher change in conceptions as a gradual process that allows varying degrees of intensity on a developmental continuum.

In a study on student teachers’ cognition development, Cabaroglu and Roberts (2000) identified 11 categories of change processes, namely, awareness, confirmation, elaboration, addition, re-ordering, relabeling, linking up, disagreement, reversal, pseudo-change, and no change. Drawing on several related studies, Tillema and van der Westhuizen (2006) proposed three criteria to promote teachers’ collaborative knowledge construction: (1) raising awareness and gaining further understanding and insights; (2) recognizing others’ viewpoints as relevant and valid to achieve perspective; and (3) involving in the group process and showing interest in using the practical results. In a longitudinal study examining the process of teacher learning, Bakkenes et al. (2010) identified six kinds of thinking processes that led to varying learning outcomes, among which three were associated with the positive aspects of learning, namely, experimenting, considering one’s practice, and getting ideas from others. Focusing on teachers engaged in a post-graduate-level teacher education course, Selvi and Martin-Beltrán (2016) found that teachers engaged in three kinds of active learning processes: externalizing their stance and challenging theory, struggling with contrasting perspectives, and re-situating theory in light of past, present, and future teaching–learning contexts. The researchers emphasized that the praxis building of teachers is dynamic, multifaceted, evolving, and negotiated in context. In a comparative study on a short-term certification program in South Korea and a comparable long-term program in the United States, Choi (2017) summarized three interrelated patterns of cognitive change, namely, capturing and repositioning their assumptions, gaining and seeking pedagogical implications, and inner conflicts and reconciliation.

It can be concluded that our understanding of teachers’ sense-making processes remains inadequate. Tillema and van der Westhuizen (2006) warned that a lack of agreement regarding the nature, process, and outcome of teacher learning would lead to less precise prospects for knowledge building in the profession of teaching. Therefore, more research is needed to capture “how teachers come to know what they know” and “how certain concepts in teachers’ consciousness develop over time” (Johnson, 2009, p.17), particularly in the context of continuing professional development. To contribute to this domain, the current study adopts a qualitative case study approach (Merriam, 2001) to explore both the outcome and the process of learning during teachers’ participation in a CPD program. We specifically focus on teachers’ changes in their conceptions of research throughout the program. The study was guided by the following research questions:

1. Will the participating teachers’ conceptions of research change over the course of the CPD program? If yes, what kind of change has taken place?
2. What are the program characteristics that contribute to the change?
3. How do the teachers reconstruct their conceptions of research during their participation in the program?

## The study

### Participants

The participating teachers in the program were 33 senior high school EFL “gugan” (literally “backbone” in Chinese) teachers, that is, outstanding teachers selected by their schools and accredited by the local Education Commission. Their participation in the program was mandatory for the renewal of the title. A total of five demographically diverse teachers were purposefully chosen as the “focal” cases (Merriam, 2001) in the current study, who exhibited a high degree of involvement in the program since the commitment to collaboration has been considered a crucial factor that may affect the results of learning (Tillema and van der Westhuizen, 2006). They were pinpointed according to the quantity and quality of their verbal contributions to the group activities after the first author calculated and reviewed the valuable insights (i.e., ideas that were related to the input of the workshop and the connections they made to their contexts) generated by each participating teacher in the first workshop, based on the teachers’ oral debriefings after group discussions and their first written reflections. The focal teachers were approached by the first researcher before the second workshop and voluntarily agreed to take part in the study. Table 1 presents the profiles of the participants.

**TABLE 1:** Participant profiles.

Name	Gender	Age	Teaching experience (years)	Educational background	Previous research experience (self-reported number of research papers written)
**Lucy**	F	30	5	MA in English literature	8
**Jane**	F	27	5	BA in English education	5
**Wanda**	F	33	10	BA in English literature	More than 10
**Frances**	F	39	18	BA in English education	more than 30
**Sunny**	F	50	33	MA in English education	more than 60

All five participants were women, whom we called Lucy, Jane, Wanda, Frances, and Sunny. They had a variety of research experiences, such as writing or publishing research papers and/or engaging in research projects. The more experienced they were, the more extensively and intensively they had been involved in research. This is understandable as engagement in research is one of the major conditions to qualify for the title of “gugan” teacher in China.

### Data collection

Data in this study included teachers’ written reflections, research proposals and manuscripts of research papers submitted in fulfillment of the requirements of the program, and interviews. After each workshop, every participating teacher was required to submit a written reflection on their learning experiences following a KWLH (what I know, what I want to know, what I have learned, and how I put it into practice) protocol. At the end of the program, the participating teachers were expected to submit a proposal on researching reading–writing-integrated EFL instruction, based on which a full-length research paper was developed within 2 months after the completion of the program. The preparations for the final research paper permeated the whole program. In each workshop, the teachers discussed the selection of research topics and the choices of research design with their peers and the teacher educator based on the workshop input (e.g., lectures and reading). By the end of the program, most of the teachers have finished their data collection. The tasks that remained after they exited the program were to analyze the data and draft the paper.

Upon the completion of the program, the first author held semi-structured interviews individually with each focal teacher. The interviews covered the following topics: (1) learning experiences in the program, (2) past experiences of research and future research plan, (3) difficulties in conducting research, (4) understanding of research, and (5) understanding of EFL reading and writing. The interview data were transcribed by a research assistant.

The data sets constructed included (1) 20 written reflections (four for each teacher), (2) five audio-recorded and transcribed individual teacher interviews, and (3) five research proposals and research papers. The multiple sources of data enabled the researchers to triangulate the data and guarantee the trustworthiness of the study (Miles and Huberman, 1994).

The researchers were aware that the use of data from interviews and program assignments would introduce accommodation bias or the possibility that the participants would make an effort to generate examples of how the program had influenced them simply to be helpful (Kennedy, 2008). Therefore, the focal teachers were informed that the purpose of the study was to help the teacher educator improve the future design of the program, and they were encouraged to objectively comment on their learning experiences. As the program was not supposed to give teachers grades, there was no conflict of interest.

### Data analysis

Multiple sources of data were first analyzed individually and then combined and reanalyzed in a qualitative manner (Miles and Huberman, 1994). The process involves three steps. First, we read through all the data to reveal salient themes and recurrent patterns, and then the data from each teacher were reanalyzed based on these themes. The coding was conducted both deductively by using *a priori* categories evolved out of the literature and the research questions, and inductively by identifying the concepts that emerged from the data. Second, we conducted a cross-analysis of data, in which meaningful themes emerging from the interview data were compared with themes emerging from teacher reflections. The themes were then triangulated with teachers’ research proposals and research papers. The cross-check produced 10 sub-themes, namely, “conceptions about the nature of research,” “conceptions about the purpose of research,” “conceptions about the relationship between teaching and research,” “conceptions about the process of research,” “peer interaction,” “reading,” “scaffolding,” “validating,” “linking up,” and “re-situating,” which were grouped into three broad categories: teachers’ conceptions of research, factors that contribute to the change, and the process of change. Based on the final themes and sub-themes, data from the five focal teachers were scrutinized to identify changes in teachers’ conceptions, which led to the creation of Appendix Table 1. Third, the final results from the multiple sources of data were cross-compared numerous times to validate the findings.

All the data were analyzed collaboratively by the two researchers. Each author first analyzed the data independently and then compared the results to enhance the credibility. The comparisons and discussions allowed the research team to agree on all the themes and quotes. Meanwhile, member checking was conducted to verify an accurate interpretation of the data, and where necessary, clarification was obtained from the teachers.

## Findings and discussion

### Changes in teachers’ conceptions of research

Data analysis has revealed that the five focal teachers reconstructed their conceptions of research in four major areas: (1) the nature of research, (2) the purpose of research, (3) the relationship between teaching and research, and (4) the process of research. Appendix Table 1 presents the teachers’ conceptions before and after the program with abridged quotes from teacher reflections and/or interviews.

First, the teachers developed a broader awareness of the different forms that research can take and the varying approaches to research. On entry into the program, the teachers viewed research as something “grand,” or academic, which should build on “sound theories and rich data,” employ “complicated statistical tools,” and produce “generalizable results.” The view aligns with a positivist notion of scientific research and resonates with the findings of previous studies conducted both in China and in other countries (Borg, 2009; Gao et al., 2011; Gao and Chow, 2012).

At the end of the program, the teachers’ understanding of the nature of research became more comprehensive. They realized that research could be “small,” or teaching-oriented; it should “stand on the solid ground of teaching” and include “studies about any aspects of teaching with various methods and tools available,” for example, action research, lesson study, and narrative inquiry. Sunny’s comments reflected the emerging conceptions:

“All that we have experienced in our everyday practice can become the subject of research. Research is to transform the phenomena that we observe into something that can better guide our teaching through rational thinking and systematic organization.” (Sunny, interview)

Meanwhile, the learning experience in the program resolved the teachers’ uncertainties about the nature of the research. For example, in her first written reflection, Jane reviewed her previous research experiences and questioned the “research” that she has done before:

“…we wrote ‘papers’ summarizing teaching almost every semester and shared them with our colleagues. I’m not sure whether these can be called research.” (Jane, reflection 1)

When she was asked the same question in the follow-up interview, Jane responded with more certainty:

“Now, I think what we have done IS research. I understand ‘research’ from two perspectives. On the one hand, research helps us find feasible ways to teach. On the other hand, it helps us solve practical problems in the classroom. In this sense, I think we do well in research because we have done a lot to improve teaching. What we lack is the ability to turn everyday practice into research.” (Jane, interview)

Second, the teachers’ conceptions about the purpose of research shifted from “instrumental” to “integral.” In line with the previous studies (e.g., Gao et al., 2011), all the focal teachers agreed that research was beneficial to their professional growth. But they engaged in research mainly to meet the administrative requirements for appraisal and promotion. As a result, they felt overburdened by all kinds of “research assignments.”

In the program, the teachers realized that research should not become an ‘extra burden.’ Both reading and doing research are to solve problems in their teaching practices. Jane’s written reflection illustrated the point:

“We seldom think about what to write until it’s almost the deadline…But now, I think, teaching comes first. When you have done something new or meaningful, you will be eager to share it with other teachers. Then you start to write. On the other hand, if you’re faced with problems, go and read the literature. You can find the solutions from other teachers’ experiences.” (Jane, reflection 3)

Sunny agreed with the idea that the purpose of doing research is to “publish,” or to “share your experiences with other people so that the whole community of teaching can reap the benefits” (interview). She argued that research should not become something that is imposed on teachers:

“Research embodies your personal feelings and emotions because you put your time and energy into it. It’s something personal, not institutional.” (Sunny, interview)

Third, along with the change in the conceptions about the purpose of research, the teachers’ views about the relationship between research and teaching have also been elaborated. Before the program, their understanding was unidirectional, mainly focusing on what research could do for teaching such as recording, sharing, and improving teaching. They thought that research was independent of teaching, so they had to “balance” teaching and research in their busy schedules. After the program, all the focal teachers agreed that “research is interwoven with the actual teaching process.” Jane reflected,

“Research is a synthesis of teaching. Teaching feeds research and research nourishes teaching. They are nurturing each other.” (Jane, reflection 2)

The idea was supported by Frances who wrote in her final reflection that “teaching is research and vice versa” and further explained the idea in the interview:

“They (teaching and research) are closely related and can never be separated. When a teacher takes a full record of her practice and analyzes it systematically, she is doing research. To research is to record, reflect and refine teaching.” (Frances, interview)

Sunny described her understanding of the relationship between research and teaching in her final reflection and used a metaphor in the follow-up interview to illustrate the idea:

“…our research is grounded in teaching and teaching enriches research. We naturally turn into a teacher-researcher.” (Sunny, reflection 4)

“Research is salt, without which teaching would be tasteless. An appropriate amount of research would make your teaching career tasty.” (Sunny, interview)

Wanda revealed the changes in her understanding of the relationship between teaching, research, and theory in her third reflection:

“I had thought that research means to apply theories to practice…I have learned that theories also come from teaching. Research helps generalize theories from teaching. It is a bridge between theory and practice.” (Wanda, reflection 3)

Fourth, by giving the teachers a thorough grounding in basic research concepts that they could draw on consistently, such as reading for relevant theories, selecting research paradigms and methods, and systematic gathering and interrogation of evidence from a range of sources, the CPD program consolidated the teachers’ view that research should be conducted systematically.

The focal teachers admitted that their previous approach to research was rather “unsystematic” and the papers they have written were “like diaries.” By the end of the program, most of the teachers were able to articulate the whole research process.

“When we teach with a purpose, or when we have a vision for our students, we begin to design specific plans. Then we implement the plan step by step. This is the process of research.” (Sunny, reflection 4)

In both written reflections and follow-up interviews, three of the focal teachers mentioned the need to “reverse” how they used to conduct research. Lucy wrote in her second reflection that research means “organization and classification of materials,” and she elaborated on the point in the interview:

“I usually put my teaching materials in different folders on my computer and label them according to the contents. For example, one folder contains my teaching plans. The other contains my students’ work. When I want to write a paper, I have to search through these folders for all the necessary materials. But now I realize that the process should be reversed. I should decide on the research topic first and develop a research plan. In this way, I will be able to determine the types of data I need for researching the topic and keep a record with a specific focus. The folders will then be organized according to research topics and data types… In our busy work, if we can spend some time sorting out and categorizing what we have done, we’ll become good researchers.” (Lucy, interview)

In the interview, Wanda described the features of the research process according to her understanding of action research cycles:

“Research is cyclical. When we discover a practical problem in our classrooms, we try to find a way to solve it. Then we identify new problems and continue the cycle. In this research process, we improve our teaching practices and deepen our understanding of teaching.” (Wanda, interview)

### Program characteristics that contribute to the change

A total of three favorable conditions have been found to facilitate teacher change, namely, peer interaction, literature reading, and teacher educator’s scaffolding. The following section illustrates how the teachers understand the three program characteristics and how they function to trigger the change.

#### Peer interaction

Both the written reflections and follow-up interviews revealed that the teachers highly valued the experiences of peer interaction because the collective discussions engaged them in reflective dialogs about their previous teaching and research experiences and their understanding of the program contents. The interaction helped externalize the teachers’ private practices and implicit personal theories, functioning as a mediator for collaborative knowledge construction. Through peer interaction, the teachers discovered alternatives to their work and gained new insights into the teaching and research of EFL reading and writing. For example, Lucy wrote in her second reflection that the discussions enriched her repertoire of teaching and enlightened her from time to time. Frances shared a similar idea:

We share our experience and practice. The discussions free our thinking which tends to be suppressed by our daily routine. It’s a collision of ideas, from which we discover similarities, look for differences, and draw inspiration. (Frances, interview)

The findings lend support to the argument developed by previous studies that the insights into the topics, generated through interaction with their peers, enable teachers to enhance or fine-tune the personal knowledge base of their work (Cochran-Smith and Lytle, 1999; Orland-Barak, 2006; Tillema and van der Westhuizen, 2006). In the present study, peer interactions allow the teachers to share different teaching styles and research possibilities, express divergent beliefs and perspectives, and search for alternatives.

In addition, the present study also found that the dialogs were particularly useful to less experienced teachers, who sought to network with their more experienced counterparts and thereby expand their social and professional connections. Jane, the youngest focal teacher in the study, reflected how the collaborative dialogs acquainted her with other teachers:

“Through the discussions, I got to know a lot of outstanding teachers in our group, who came from different schools in our district. They will become my resources. In the future, I know whom I can turn to for help if I encounter any problems.” (Jane, interview)

Borg (2014) pointed out that the opportunity to “meet, talk to, exchange ideas with, and learn from other ELT professionals” would make an important contribution to teachers’ professional development (p.39). This is especially true in China where “guanxi,” a form of social connectivity deeply entrenched in culture involving the continuous exchange of favors among individuals, exerts an intangible influence over individuals’ lives and social practices (Bian, 2018). Having a good “guanxi” with these outstanding teachers of a higher level or ranking (e.g., principals or head teachers) means easier access to highly competitive prizes, honors, and more chances for professional development. By connecting “gugan” teachers in possession of both professional expertise and professional resources, the CPD program helped the younger teachers weave and expand their “guanxi” network in the professional community, resulting in more possibilities to achieve greater success in their future careers.

#### Reading of research studies

This study also found that reading research papers created a positive effect on teacher professional development. In the current CPD program, reading materials were distributed in electronic format several days before each workshop so that the teachers could read by themselves in their private time. During the workshops, the teachers read the materials again in groups with the help of the teacher educator, resulting in three apparent benefits. First, reading enhanced the teachers’ research literacy. In the following excerpt, Jane discussed how the reading of research studies acquainted her with the basics of good research:

“They (the papers) showed how research can be done, how we can select the participants and collect the data…I got more familiar with the structure of an academic paper. When I’m to write a paper in the future, I will probably read these papers again.” (Jane, interview)

Second, reading encouraged the teachers to analyze their research practices based on the new meanings derived from other teachers’ research perspectives. Sunny described how the reading restored her professional confidence and reduced the feeling of uneasiness that surfaced in her classroom experimentations:

“We’re now incorporating the reading of original English novels in our class. But we’re not sure whether the new method would work. Sometimes we feel uneasy because we have to bear the pressure coming from the students, the parents, and the school administration. When I read about the same practice researched by other teachers in their published papers, I feel reassured. I think we’re probably walking down the right path and simply need more persistence.” (Sunny, interview)

Third, reading prompted the teachers to reflect on their classroom realities and apply the research findings to their contexts. The teachers thought that most of the research studies that they have read in the workshops were practically oriented and therefore “attractive” to them. After reading these papers, they were “eager to apply the results” to their classrooms. Wanda expressed her interest in experimenting with continuation writing tasks in her reflection:

“I think the continuation writing tasks used after reading could be a very useful way to integrate writing with reading. My students are always frustrated with English writing. If I ask them to continue a story after they have finished reading it, they will probably be interested.” (Wanda, reflection 3)

Gao and Chow (2012) argued that teachers need to learn from reading other teachers’ research narratives, the research procedures that were adopted, and the data that were collected in the process. The rich understanding derived from reading inspired the teachers to reflect on their contexts and determine whether and how they can utilize the new information. This study proves the positive effect of reading on helping teachers reconstruct their conceptions of research, as well as their teaching and research practices.

#### Teacher educator’s scaffolding

This study found that the teacher change is related to the teacher educator’s scaffolding in both peer interaction and reading. On the one hand, the focal teachers highly appraised the teacher educator’s participation in their collaborative discussions. During the discussions, the teacher educator employed mediational strategies such as asking confrontational questions to push the teachers to articulate their ingrained assumptions, using direct questions to uncover what might have been concealed from the teachers and offering comments to expose the teachers to various perspectives. The dialogic interactions triggered the teachers to have in-depth reflection and consequently support their collaborative knowledge (re)construction.

The focal teachers thought the feedback offered by the teacher educator during their group discussions was “timely,” “engaging,” and “stimulating,” helping them detect the connection between practices and theories and rethink the relationship between teaching practices and doing research. Jane reflected on the feedback that she received after group discussions:

“At the beginning, it seemed like casual sharing of teaching practices in groups. But when we finished our oral reports, she (the teacher educator) asked several questions about our ideas and gave some comments. She helped us examine our classroom practices from various researchable perspectives. Then we got a lot of new ideas about research and suddenly realized that there could be so much to write about.” (Jane, reflection 2)

In the follow-up interview, Sunny commented on the timing of the teacher educator’s participation:

“Most of us are experienced teachers. Some of the ideas (raised by the teacher educator) are not strange to us. But the timing was perfect. They were offered during or after our discussions when we were actively engaged. We could immediately understand how they can be practically applied to our teaching and research. The suggestions and comments made the whole discussion more fruitful and enlightening.” (Sunny, interview)

It is therefore evident that rather than imposing a preferred new practice on the teachers, the teacher educator paved a new way of seeing, understanding, or interpreting the familiar ideas, rendering them more applicable to the teachers’ contexts.

On the other hand, scaffolding was provided when the teacher educator guided the teachers through their reading of the selected research studies. Instead of plunging the teachers into a sea of literature, the CPD program adopted a guided reading procedure, in which the teachers read and discussed the literature in groups and the teacher educator provided feedback tailored to the needs of the ongoing discussions in the forms of summarizing, questioning, clarifying, and critiquing. With the support of the teacher educator, reading became easier and more rewarding. For example, Lucy discussed her experience of the guided reading:

“When I read the papers at home, I found it very difficult to understand, particularly the theoretical concepts and the internal logic of writing. But with the help of the teacher educator, I came to understand them.” (Lucy, interview)

The effect of the guided reading can be triangulated with Lucy’s description of her mentor during the interview, in which she attributed the master teacher’s skillful lesson design to her “PCK”—a term Lucy has read about in one of the papers:

“My mentor plans her lesson in such a structured way that her class is always enjoyable to students…I think it is the PCK that enables her to do so.” (Lucy, interview)

The previous excerpt proved that Lucy not only understood the academic term in the paper but also was able to reflect on her own professional life in the professional discourse. The results indicated that with proper guidance and support, reading can become less cumbersome and more relevant to the teachers.

In the present CPD program, the teacher educator acted as a co-thinker, mediator, and critical friend to scaffold the collaborative efforts of the teachers. With the support and the prompt, the teachers were aided in reflective thinking to articulate their implicit assumptions about teaching and research, scrutinize the different facets of their practices, and explore the perspectives that they might otherwise not be able to see.

Effective participation of teacher educators has long been recognized as a vital requirement of professional programs that aim to transform teachers in both conceptions and practices (Hayes, 2019). Ingvarson et al. (2005) highlighted that timely and insightful feedback on what one is doing or has done is crucial to the reflection on one’s practices and the development of understanding. The current findings confirmed the argument developed by many researchers that continuing professional development characterizing teacher collaboration, reading, and mentoring can function more successfully than those one-shot courses targeting the transfer of certain content to teachers (Mann, 2005; Borg, 2015). The results also highlighted the value of professional support frameworks designed around a collaborative discussion component in CPD programs and accentuated the mediational role played by teacher educators in assisting teachers to develop their practical wisdom (Orland-Barak, 2006; Luneberg and Korthagen, 2009; Sedova, 2017).

### Processes of change in teachers’ conceptions

Concerning how teachers reconstructed their conceptions of research, this study found that the focal teachers engaged in three active processes of knowledge (re)construction throughout the CPD program: (1) validating one’s practices, (2) linking others’ perspectives up to one’s own, and (3) re-situating research in light of one’s current practice. The three types of mental activities, varying in the depth and breadth of thinking, share a common denominator, that is, the teachers’ reflection on their past and present experiences in connection with the program input.

#### Validating my practices: I’m doing right!

The knowledge (re)construction at the lowest level is to validate the practices according to the information obtained in the program, a process in which all the focal teachers have taken to confirm their stance. Belcher (2007) pointed out that teachers are sometimes faced with making pedagogical decisions without complete confidence in their efficacy. The CPD program provided a framework for the teachers to reexamine their experience-based intuitive teaching and research.

In the interviews, the teachers claimed that they “became more confident” as they found other teachers doing “similar things” in different contexts and “explored more possibilities for future research.” For example, Lucy discussed how the reading of research studies prompted her reflection on practice and triggered her interest in conducting research:

“I have incorporated the reading of original novels in my class for a whole year and published 2 articles. In this program, we have read several articles about extra curriculum reading. Some were written from perspectives similar to my own. Others were from different perspectives… I’ve got many ideas for further study.” (Lucy, interview)

This pattern of thinking has also emerged from the case of Sunny. For many years, Sunny has implemented inquiry-based learning in her teaching or reading. For example, after finishing a textbook unit about the topic of success, she asked her students to read some famous people’s success stories and write group reports on the elements that lead to their success. In this learning process, she acted as a facilitator to help her students select the reading materials and write the reports. During the discussion of potential research topics, Sunny shared her practice with other teachers and was encouraged by the teacher educator to research it. The following excerpt is her reflection on the learning experience:

“I’ve met many objections these years. They said it was too time-consuming. But I continued because my students liked it. When I shared this practice in our discussion, the teacher educator suggested that I do some research on it. She recommended some books about inquiry-based learning. I feel more confident now for I’m doing right!” (Sunny, interview)

Paran (2017) contended that research-oriented or evidenced-based CPD programs can correct unreliable intuition and experience and break the vicious circle of received wisdom. Our study aligns with this argument and further proposes that such a CPD program could connect teachers’ intuitive teaching to research evidence and provide the necessary professional and emotional support to validate their professional wisdom.

#### Linking others’ perspectives up to my own: I can also do that!

At a higher stage of knowledge (re)construction, the focal teachers utilized the social context provided by the CPD program to locate their positions not only in their own experiences but also in the experiences of others. With the diversified perspectives afforded by the peers, the teacher educator, and the authors of the selected reading, the teachers engaged in an active synthesis of information, sorting out the relevant viewpoints and connecting those with their contexts, based on which the teachers reconstructed their conceptions and practices. For example, both Jane and Frances reported that they modified their teaching practices according to what they had read and/or discussed. Frances conducted a study on the effectiveness of the new practice in her final research paper.

Belcher (2007) proposed that when teachers read published research articles, they bring not only a fund of knowledge based on prior teaching and research experience but also the current awareness of their local situations, all of which place them in a strong position to judge the relevance and transferability of the pedagogical implications of those studies. The current findings further testified that teachers are inclined to experiment with the relevant ideas in their classrooms and transform their teaching and research practices accordingly.

Moreover, the linkup process also resulted in the incorporation of new research tools into the teachers’ repertoire of research practices. In the interviews, some of the teachers explicitly expressed their interest in the newly acquainted research methods. For example, after reading some research studies conducted with action research and listening to the teacher educator’s lecture about the method, Lucy wrote in her reflection,

“I think it’s a very useful method for teachers. When I’m faced with a problem, I can use action research to solve it.” (Lucy, reflection 4)

In the discussion of research proposals when Sunny shared her experience of the inquiry-based reading and writing project, the teacher educator introduced the method of narrative inquiry and suggested that in addition to drafting lesson study reports and evaluating the effect of the project on students with experiments, the teacher could use narrative inquiry to record her experiences in the implementation of the project. Sunny reflected on the episode in the interview,

“Telling stories is also research. This is new to me. I have learned a lot from this experience (inquiry-based learning). I think I can write my own story and share my experiences with others.” (Sunny, reflection 3)

The current findings confirm the argument that the teachers are interested in concrete ideas, rather than abstract or theoretical terms, and they value the research works that can be applied to their professional contexts (Guskey, 1986; Belcher, 2007; Macalister, 2018; Avidov-Ungar and Herscu, 2020). Although the teachers’ emerging interest in the new perspectives might not necessarily lead to their actual “experimentation,” they at least learned to take ownership of these new ideas when they made conscious efforts to connect them with their contexts. As Guskey (1986) has posited, when teachers are intrigued enough to try the new practices and leave the program with a ‘Well, let’s see’ attitude, the change process starts (p.9).

#### Re-situating research in light of my current practices: I can do more!

As new understanding about research emerged, the teachers took a further step to reassess the research value of their daily work and direct their research efforts in light of what they are currently doing in the classrooms. It is in this process that the teachers recognized that research should be hinged on teaching, and the everyday practices that they have taken for granted deserve more thorough investigations.

All the focal teachers reported their experiences of reflecting on alternative research perspectives based on their current practices. Some were poised to “aim higher” to “plan the teaching from the perspective of research.” In the following excerpt, Frances discussed how she decided to design a research study based on what she was doing in her classrooms:

“I tried extra-curriculum reading at the beginning of this semester. My students have read three books by now. They did all kinds of after-reading activities, for example, rewriting the ending of a story, or changing the story into a stage play. Now I think I can do more!. Maybe I can design a questionnaire to examine my students’ attitudes, and collect their assignments to evaluate the effect of such reading on writing. There are many research perspectives.” (Frances, interview)

In the following excerpt, Lucy discussed how the reading of an article about “classroom silence” triggered her interest to further probe the issue in her classrooms:

“I have experienced “classroom silence” many times. The article analyzed some of the reasons for silence but didn’t talk about how to solve the problem. I think I will review my cases of classroom silence and think about the solutions. Maybe I can write an article on this.” (Lucy, interview)

Kirkwood and Christie (2006) argued that teacher research in the context of CPD should seek to capture the uniqueness of the practitioners’ particular professional context. The current CPD program provided a chance for the teachers to critically analyze their work with the available research tools and derive research ideas from their classrooms. As a result, the teachers became more “research-minded.”

The current findings support the recent trends in teacher education toward practice- and inquiry-oriented collaborative models of CPD and prove the positive role of the CPD program in (re)shaping teachers’ conceptions of research. However, it would be too soon to conclude that the CPD program has led or will lead to changes in teachers’ conceptions. What can be certain is that the learning opportunities created by the program activities, in particular, discussions and the reading papers guided by the teacher educator, constitute an important facet of “the change environment” that affords teacher growth (Clarke and Hollingsworth, 2002, p.954). The catalysts that act upon the environment and accelerate the transformation are teacher reflection and their willingness to learn.

On the one hand, teacher reflection mediates the teachers’ change process. The conversations with peers, the reading of professional publications, and the input from the teacher educator provided the teachers with a new stimulus to articulate, negotiate, and develop a new understanding of teaching and research. As Tillema and van der Westhuizen (2006) have pointed out, teacher collaboration, together with reflection, is a central feature of collaborative knowledge construction.

On the other hand, teachers’ willingness to learn, that is, a psychological state that “involves a desire to learn, experiment, and see or do something that has not been seen or done before” (Van Eekelen et al., 2006, p. 411), triggers teacher reflection and sets the wheels of teacher change in motion. Researchers have proved that teachers with strong wills to learn would be more likely to challenge their existing knowledge, think up and try out new actions to explore the newly observed phenomena, test the tentative understanding, or affirm the moves that they have invented to change things for the better (Oosterheert and Vermunt, 2001; Van Eekelen et al., 2006). Hayes (2019) pointed out that CPD is likely to succeed when teachers are willing to engage in CPD activities that are mandated and actively seek out opportunities to improve their practice in personally meaningful ways. The three ways of knowledge (re)construction identified in this section are indicative of the teachers’ self-regulated reflective thinking, which is driven by a strong will to learn and eventually leads to optimal learning results.

## Conclusion

The present study explored both the outcome and process of teacher learning in a CPD program locally developed as part of the routine EFL teacher development initiatives. The findings contribute to the body of work that has called for more coherent programs of teacher learning in design and implementation and demonstrate how opportunities for teacher learning can be built into CPD programs to achieve beneficial effects. The present research offers two implications for continuing professional development.

First, CPD programs that characterize features such as peer interaction and literature reading call for deep involvement of teacher educators. The guidance and feedback offered by a teacher educator or mentor could facilitate teachers’ information processing and support their collaborative knowledge construction. Second, teachers should be motivated to learn by program contents that are closely related to their professional lives. Once teachers get interested in the new ideas, they will assume an active role in the program activities and reap their benefits.

Despite the insights offered, the current study has limitations. The issue of sustainability is crucial to any CPD program if it targets teachers’ long-term development. Therefore, more longitudinal studies are needed to follow up with the teachers in their actual research engagement to capture the trajectories of learning after they have exited the program and offer more insights into how they utilize what they have obtained from the program to inform their teaching and research. Moreover, the “idiosyncrasies” of participant selection cannot be neglected when the results are to be applied to other contexts since all the focal cases are outstanding teachers with extensive research experiences, high reflexivity, and a strong desire to learn, which enabled them to fully utilize the learning opportunities afforded by the CPD program. Future CPD programs might consider revising the current program framework to satisfy the needs of target teachers. It is also unfortunate that the study was not able to involve male teachers in the focal cases to provide a more diversified perspective of teacher learning and teacher change.

## Data availability statement

The raw data supporting the conclusions of this article will be available upon request.

## Ethics statement

This study was reviewed and approved by Beijing Foreign Studies University. The participants provided their written informed consent to participate in this study.

## Author contributions

YK did the initial data analysis and drafted the literature review and findings. LY created the context for data collection and finalized the manuscript. Both authors contributed to the article and approved the submitted version.

## Appendix

**TABLE 1:** Summary of changes in teachers’ conceptions of research.

Name	Sub-categories of conceptions of research	Before the program	After the program
**Lucy**	Nature of research	Research should be scientific.	Research does not need to be very theoretical. Research should be grounded in teaching.
	Purpose of research	I wrote papers to record the new practices in my classroom.	We can study both new practices and classroom routines to improve teaching.
	Relationship between teaching and research	Research helps record my experiments in teaching.	Research is about every aspect of teaching. Research and teaching are interwoven.
	Process of research	The papers I have written were not very professional. I look for materials when I’m to write a research paper.	My old way should be reversed. Research starts from a purposeful collection of materials
**Jane**	Nature of research	Not sure what is research. What I have done before was probably not real research. Research topics should be grand.	What I have done IS research. It helps find feasible ways to teach and solve practical problems. Research topics can be small.
	Purpose of research	We wrote papers to meet all kinds of requirements.	We write to share new practices with others and find solutions from reading research.
	Relationship between teaching and research	Research is for recording and reflecting on teaching.	Research and teaching are nurturing each other.
	Process of research	Research should be systematic. My research is only a summary of teaching.	We can choose a topic, select a method, and collect data in the process of teaching.
**Wanda**	Nature of research	Good research needs (statistical) data. Research should produce generalizable results.	To do research is to study our practices. There are many research methods.
	Purpose of research	As a teacher, I am supposed to do research. Doing research will improve teaching.	To do research is to solve our problems in teaching.
	Relationship between teaching and research	Research means to apply theories to practice.	Theories also come from teaching. Research helps generalize theories from teaching.
	Process of research	We have a lot of data but do not know how to develop them into real research.	Research is a process of discovering problems, solving problems, and rediscovering new problems.
**Frances**	Nature of research	Research needs theories and data. We use statistics to analyze data.	Research means the studies about teaching with various methods and tools available.
	Purpose of research	To do research is to share my experiences with others.	To do research is to record, reflect, and refine teaching.
	Relationship between teaching and research	Research can improve teaching. I have to strike a balance between teaching and research.	Teaching and research are closely related and inseparable.
	Process of research	Research should be carried out in a step-by-step manner.	Research requires systematic planning and data collection. We plan the research before we teach.
**Sunny**	Nature of research	To do research is to have experiments. Research is data-driven.	Everything about teaching can be researched. Research takes many forms. It can be narrative.
	Purpose of research	The school wants us to do research. It is part of our job. We do experiments in classrooms to improve teaching.	To research is to publish and to share. Research is personal, not institutional.
	Relationship between teaching and research	Research helps improve teaching.	Research is grounded in teaching and teaching enriches research.
	Process of research	Research needs systematic planning.	Research requires rational thinking and systematic organization. Research is to implement the plan step by step.
